# *PURA*-Related Neurodevelopmental Disorders with Epilepsy Treated with Ketogenic Diet: A Case-Based Review

**DOI:** 10.3390/genes15070848

**Published:** 2024-06-27

**Authors:** Raffaele Falsaperla, Vincenzo Sortino, Marina Antonietta Schinocca, Gaia Fusto, Roberta Rizzo, Chiara Barberi, Martino Ruggieri, Xena Giada Pappalardo

**Affiliations:** 1 Neonatal Intensive Care Unit and Neonatal Accompaniment Unit, Azienda Ospedaliero-Universitaria Policlinico “Rodolico-San Marco”, San Marco Hospital, University of Catania, 95123 Catania, Italy; 2 Unit of Pediatrics and Pediatric Emergency, Azienda Ospedaliero-Universitaria Policlinico “Rodolico-San Marco”, San Marco Hospital, 95123 Catania, Italy; sortino.vinci@gmail.com; 3 Department of Medical Science-Pediatrics, University of Ferrara, 44124 Ferrara, Italy; 4 Postgraduate Training Program in Pediatrics, Department of Clinical and Experimental Medicine, University of Catania, 95123 Catania, Italy; marinaschinocca@gmail.com; 5 Department of Biomedical and Biotechnological Sciences (BIOMETEC), University of Catania, 95123 Catania, Italy; gaia.fusto@studium.unict.it (G.F.); rizzo.roberta@studium.unict.it (R.R.); xena.pappalardo@irib.cnr.it (X.G.P.); 6 Postgraduate Training Program in Pediatrics, University of Palermo, 90133 Palermo, Italy; chiara.barberi@hotmail.com; 7 Unit of Clinical Pediatrics and Unit of Rare disease AOU “Policlinico”, PO “G. Rodolico”, University of Catania, 95123 Catania, Italy; m.ruggieri@unict.it; 8 National Council of Research, Institute for Research and Biomedical Innovation (IRIB), Unit of Catania, 95123 Catania, Italy

**Keywords:** ketogenic diet (KD), *PURA* gene, seizure, sialorrhea, in silico analysis, whole exome sequencing (WES)

## Abstract

*PURA* syndrome is a congenital developmental disorder caused by de novo mutations in the *PURA* gene, which encodes a DNA/RNA-binding protein essential for transcriptional and translational regulation. We present the case of an 11-year-old patient with a de novo frameshift variant in the *PURA* gene, identified through whole exome sequencing (WES). In addition to the classical *PURA* deficiency phenotype, our patient exhibited pronounced sialorrhea and seizures, which were effectively treated with the ketogenic diet (KD). Our integrative approach, combining a literature review and bioinformatics data, has led to the first documented clinical case showing improvement in both sialorrhea and seizures with KD treatment, a phenomenon not previously reported. Although a direct relationship between the de novo *PURA* mutation and the KD was not established, we identified a novel frameshift deletion associated with a new clinical phenotype.

## 1. Introduction

*PURA* syndrome, or also known as *PURA*-related neurodevelopmental disorder, has recently been described as the neurodevelopmental disorder with neonatal respiratory insufficiency, hypotonia, and feeding difficulties (NEDRIHF; OMIM#616158) [1]. It is a rare autosomal dominant disease, with fewer than 150 cases reported in the medical literature. The condition is characterized by a spectrum of multisystemic phenotypes, including global neurodevelopmental disorder [2] The main features in neonates with *PURA* syndrome are feeding difficulties (97%), hypotonia (82%), primary apnea or hypoventilation (60%), and drowsiness (50%) [3]. All patients exhibit moderate-to-severe intellectual disability (100%), and epilepsy (50%) is often refractory to medical intervention, with some patients never achieving seizure freedom despite treatment efforts [1]. According to the latest NICE guidelines, the treatment of drug-resistant epilepsy includes the possibility of using the ketogenic diet (KD) for seizure control [4].

We describe a patient with *PURA* syndrome with a novel frameshift mutation, detected by WES, who responded positively to ketogenic diet (KD) treatment, including resolution of sialorrhea and reduction in seizure frequency. We also collected data on the reported *PURA* cases treated with the KD in order to correlate the pathogenic variants with the respective outcomes of KD therapy, if applied.

In order to evaluate whether the clinical phenotype could be linked to the type and position of the identified mutation, we compared the 3D model of the native and mutant proteins based on the available crystal structure of human Purα.

## 2. Materials and Methods

### 2.1. Review Search Strategy

A literature review was conducted by collecting clinical trials, primary research, and reviews from online bibliographic databases (MEDLINE, Embase, PubMed, Cochrane Central, and Scopus) covering the past ten years. The key search derived from the medical subject headings (MeSH) and included “*PURA* syndrome”, “*PURA*-related neurodevelopmental disorders”, “neurodevelopmental disorder with neonatal respiratory insufficiency, hypotonia, and feeding difficulties (NEDRIHF)”, as well as terms related to ketogenic treatments, such as “ketogenesis”, “ketogenic diet/treatment/therapy”, “ketone bodies”, or “fatty acid-derived ketone bodies”. Genetic studies, case reports, and review articles were manually examined and included in the present reference list. After removing duplicate records, the main search results were compiled for inclusion in the study.

### 2.2. Genetic Testing

Genomic DNA was extracted from the child and parents to perform the clinical exome sequencing (Trio-WES). The exonic regions and flanking splice junctions of the genome were captured using the SureSelect Clinical Research Exome (Agilent Technologies, Santa Clara, CA, USA). Massively parallel (NextGen, Atlanta, GA, USA) sequencing was carried out on an Illumina system with 2 × 150 bp paired-end reads. The median depth of coverage exceeded 199× for all three exomes, with over 98.6% of the exome covered at >10×. Reads were aligned to human genome build GRCh38/UCSC hg38 and analyzed for sequence variants. The analysis considered the minor allele frequency (MAF) in population databases (GnomAD, EVS, and 1000 genomes), the presence of variants in databases of medically relevant variants (ClinVar, Human Gene mutation Database), the predicted impact on coding sequence, and the mode of inheritance by in silico tools (Varsome, PolyPhred, Sift, Provean, MutationAssessor, MutationTaster v.2021). The interpretation of variants was conducted according to the guidelines of the American College of Medical Genetics and Genomics (ACMG) [5] and Association for Clinical Genomic Science (ACGS) recommendations [6]. Variant calling for Single-Nucleotide Variants (SNVs) and small insertions/deletions (indels) was performed using Illumina’s DRAGEN v3.2.8 software, while structural variants, including Copy-Number Variants (CNVs), were analyzed using the ExomeDepth algorithm [7].

### 2.3. Protein Modelling

To reconstruct the three-dimensional structure of the protein, we utilized MODELLER v.10.1 software (salilab.org/modeller/; last accessed on 15 April 2024) [8]. This computational simulation was based on statistical parameters derived from the GA341 (Genetic Algorithm 341) and DOPE (Discrete Optimized Protein Energy) scores, following the program’s instructions (refer to Table S1). The modelling process employed the crystal structure of human Purα (fragment Glu57-Glu212, PUR repeats I and II) from RCSB PDB (www.rcsb.org; last accessed on 15 April 2024) (PDB ID: 8CHW) [7]. The optimal protein model was visualized using Visual Molecular Dynamics (VMD), v.1.9.4 (ks.uiuc.edu/Research/vmd/; last accessed on 15 April 2024).

## 3. Results

### 3.1. Clinical Case Presentation

We report the case of an 11-year-old boy, the first child of healthy unrelated parents, who suffers from tetraparesis, severe intellectual disability, epilepsy, behavior disorder, and sleep disorder due to *PURA* syndrome. He was delivered at 41 weeks of gestation after normal pregnancy. His birth weight was 3150 g, with a length of 49 cm, a cranial circumference of 35 cm, and an APGAR score of 9/9. At three days old, the parents and physician noted hypotonia and feeding difficulties, although these issues did not affect his growth or overall clinical condition. At one month old, he was admitted to a Neonatal Intensive Care Unit and Neonatal Accompaniment Unit (NICU) due to epileptic neonatal seizures. These seizures were characterized by generalized hypotonia, desaturation and eye deviation, and they were successfully controlled with phenobarbital. A brain MRI, initially performed at that time and repeated at age seven, consistently showed minimal abnormalities, including thinning of the corpus callosum and diffuse prominence of periventricular perivenular spaces. An EEG revealed bilateral frontal abnormalities that were inconsistent with his stage of psychomotor development. He remained limited to lallation and did not progress to verbal speech. He never achieved the milestones of standing and walking. At seven years old, after a completely seizure-free period, he developed seizures again. These were characterized by flexion spasms (30 episodes/day) of the upper limbs and hypotonia of the neck with loss of consciousness. He was started on valproate but discontinued the antiseizure medication (ASM) after six months because of severe side effects. He was started on levetiracetam for the recurrence of seizures, but there was no improvement in seizure activity. At the age of 11 years, the diagnosis of *PURA* syndrome was confirmed through trio whole exome sequencing (Trio-WES), which revealed a de novo frameshift variant (c.399_400dup; p.Gln134ProfsTer92) of the *PURA* gene in the proband. This was likely pathogenetic according to genetic guidelines for variant interpretation and classification [5,6].

For poor seizure control, the ketogenic diet (KD) was initiated in accordance with the National Institute for Health and Care Excellence (NICE) Clinical Guidelines for Epilepsies in patients resistant to 2/3 ASM [4]. Prior to initiation of the KD therapy protocol, all potential contraindications to the KD, such as metabolic diseases, cardiac and/or renal dysfunction, and liver disease, were thoroughly screened for through appropriate laboratory and/or instrumental investigations [9]. Therefore, the KD therapy commenced at a ratio of 1:1, with subsequent weekly increments (2:1, 3:1 weekly) until the target ratio of 4:1 was reached. The KD showed beneficial outcomes in the management of sialorrhea, with complete resolution observed after one month of therapy. Additionally, a significant improvement in seizure control was observed, with a 50% reduction in seizures after two months of therapy, as reported in the parental diary. Unfortunately, after three months, the onset of adverse effects was observed, including marked weakness and fatigue, and the patient did not respond to fluid therapy and/or alterations in ketogenic food types.

### 3.2. Mutational Analysis

WES analysis identified a novel de novo frameshift variant (c.399_400dup; p.(Gln134ProfsTer92) in the *PURA* gene (NM_005859.5) on chromosome 5 (GRCh38) .140114580_140114581. This variant was classified as deleterious based on ACMG/ACGS criteria codes (PS2, PM2, PM4, and PP3) according to version 3 of ACMG/ACGS guidelines. Further details can be found on ClinGen’s website (www.clinicalgenome.org/affiliation/50014/docs/assertion-criteria; last accessed on 15 March 2024) [10]. The variant, with a minor allele frequency of 0 in gnomAD, leads to a truncated protein due to a reading frame shift, resulting in a premature stop of codon 92 amino acids downstream of residue 134. No additional pathogenic or likely pathogenic mutations were detected.

### 3.3. Computational Protein Modeling

To analyze and predict the effect of genetic variation on protein structure, we compared wild-type and mutated proteins. The 3D model of altered Purα computed by MODELLER v.10.1 software showing the unstructured protein folding was compared with the native protein (Figure S1).

### 3.4. Literature Review: Patients with PURA Syndrome Treated with KD

The literature review revealed only ten cases of *PURA* syndrome treated with the KD. Among these, positive effects on seizure reduction were described in only two cases. Unfortunately, the data are incomplete and contain many gaps, especially regarding treatment, side effects, and follow-up (see Table 1).

## 4. Discussion

*PURA* syndrome arises from variants in the *PURA* gene (purine-rich element binding protein A; OMIM#600473), located at chromosome band 5q31. This gene encodes a single exon, which encodes a single-exon transcript under the complex control of three promoters [12], resulting in a widely expressed and highly conserved 322 amino acid protein, Pur-α. It plays pivotal roles in gene regulation, such as performing DNA replication, transcription, mRNA transport, and translation [13,14]. Additionally, it is involved in numerous postnatal neurological processes, namely, neuronal development, proliferation, and dendritic maturation [15,16]. Structurally, Purα contains an N-terminal glycine-rich region, three Pur repeats (I–III), and a C-terminal glutamine–glutamate-rich domain. According to evidence, the higher frequency of pathogenic variants is detected at the N-terminus and within the Pur domains [12,16].

To date, approximately 65 de novo mutations have been identified in the 5’ end of *PURA* [3]. Of these, frameshift mutations causing a premature termination codon (PTC) and, accordingly, protein-truncating variants (PTVs) are emerging as the most common pathogenic variants [2]. As aberrant transcripts with PTC are eliminated by the mRNA surveillance system of nonsense-mediated decay (NMD), it has become increasingly apparent that *PURA* is one of the most enriched genes in PTVs that escape NMD [17]. However, recent evidence suggests that the analysis of the genotype–phenotype correlation is certainly not straightforward, and it may sometimes yield inconclusive results due to the high individual-level heterogeneity of affected patients [1]. Therefore, recognizing the phenotype of *PURA* syndrome in daily clinical practice is challenging. Whole exome sequencing (WES) provides an effective diagnostic tool to identify pathogenic genetic variants in patients with *PURA* [12]. Despite its significance, the role of *PURA* remains incompletely understood. This is due to the challenges associated with determining the gene interaction network in which it participates under various cellular conditions, in both healthy and diseased individuals. Understanding the effects of phenotypic variation involves elucidating differences in genetic and biological (environmental) mechanisms between the mutated and wildtype Purα. This includes investigating changes in intramolecular structural domains and protein–protein interactions, the modulation of gene expression, and the activation of diverse functional pathways. One such pathway is the escape from the nonsense-mediated decay (NMD) process [16,17,18].

Mutations have been identified in the 5’ end of *PURA* [3], resulting in a range of symptoms that almost always include neurodevelopmental delay, intellectual disability, hypotonia, and epilepsy [1,2]. It is worth noting that mutations are classified into different classes (A–E) based on their location within the protein [1]. However, recent evidence has highlighted the difficulty of analyzing genotype–phenotype correlations, which may sometimes appear inconclusive due to the elevated individual-level heterogeneity of affected patients [1]. 

In our case, WES data detected a new pathogenic frameshift variant, c.399_400dup (p.Gln134ProfsTer92), which was associated with the strongest pathogenicity according to ACMG/ACGS criteria (PS2, PM2, PM4, PP3). This variant maps within the Pur II region, responsible for the sequence-specific DNA/RNA-binding domain [13], and it belongs to class A1 according to the *PURA* mutation classification of Reijnders et al. [1] It compromises both the N-terminal and C-terminal PUR domains. Consistent with this, our predictive 3D model showed the helix destabilization and the misfolding of the Purα-altering variant (Figure S1), which is likely to disrupt RNA binding and dimerization functionalities. The stability of Purα is established by the intramolecular interaction between the α-helices of Pur I and Pur II as recently demonstrated [15]. The severity of the mutation does not necessarily correlate with the clinical response of patients positively treated with the KD. In theory, all nonsense and frameshift mutations leading to PTCs are inherent targets of the NMD mechanism, which represents a quality control mechanism that targets and rapidly degrades aberrant mRNAs carrying PTCs [19]. Recently, about 133 genes, including *PURA*, have been shown to be enriched in aberrant NMD-escaping frameshifted transcripts [17].

In the literature, only ten patients undergoing KD therapy are documented with comprehensive information regarding gender, type of genetic mutation, clinical features, age of onset and type of seizures, treatments administered, and the effects (positive/adverse) of the KD (Table 1). Of interest are three cases analyzed in the study by Reijnders et al., two of which also had sialorrhea [1]. Despite compliance with the KD in all cases, there is no clear evidence of beneficial effects or definitive data on therapy outcomes. However, it is noteworthy that none of these cases showed negative effects, prompting the continuation of the KD [1]. The patient analyzed in Mayorga et al. showed no beneficial effects on seizure outcomes despite adherence to the KD [11]. In addition, the effects on sialorrhea remains unknown, as this symptom was not addressed in the study [11]. The KD failed to improve symptoms in four of the six patients analyzed in the study by Johannesen et al. [2]. In these patients, sialorrhea was not described, leaving the effect of ketogenic therapy on this symptom unknown. In the other two cases, a reduction in seizures was observed, but there is no evidence regarding the impact on feeding difficulties [2].

Of these, the 50% of the mutations identified in patients with *PURA* syndrome treated with the KD (Table 1) are frameshift mutations (p.Ser43Alafs*34 [1]; p.Gly33Alafs*45; p.Leu54Cysfs*24; p.Leu148Trpfs*77 [2]; and p.Ile196fs*29 [11]), emphasizing the possible role of NMD in influencing the disease’s expression. The inability to remove mRNAs containing PTCs appears to play a role in influencing phenotypic heterogeneity by generating abnormal proteins, which can harm cells through dominant-negative or gain-of-function effects. Therefore, eluding nonsense-mediated decay (NMD) does not consistently lead to a more severe phenotype [19]. Accordingly, the phenotype outcome of our patient could be affected by the activity of the mutated protein, but also from the activation of the non-canonical NMD pathway.

The available experimental data on *PURA* and the cases reported in the literature do not allow us to predict or propose a relationship between the identified variant and the response to the KD. While we acknowledge the limitations of our study, which warrant further investigation from a biochemical perspective, the results obtained can be considered as a valuable contribution to expanding the set of known pathogenic variants associated with *PURA* syndrome and to expanding research on a new rare phenotypic trait that is KD-related [4]. Our findings may be associated with a severe clinical phenotype characterized by pharmacoresistant epilepsy. KD therapy has shown significant efficacy in seizure management and has been observed to have a favorable effect on the control of sialorrhea. Remarkably, this phenomenon has not been observed in other reported patients, making our case the only one described in the literature.

Our patient’s case illustrates how adherence to the KD alleviated sialorrhea and feeding difficulties, consequently enhancing his quality of life. As shown in Table 1, it is evident that adverse effects of the KD, such as marked weakness and fatigue, were only documented in our case; other patients did not need to discontinue ketogenic therapy. Currently, there are no reported data on the efficacy of the KD on sialorrhea, a symptom that is almost ubiquitous in patients with *PURA* syndrome. However, in our case, treatment with the 4:1 KD resulted in complete remission of sialorrhea, accompanied by a significant enhancement in overall well-being. This likely attributed to the KD’s modulation of potassium influence within the salivary gland acinar, achieved through direct action being taken on potassium channels and indirect modulation of dopaminergic tone. Such modulation is crucial for proper salivary fluid secretion [20,21,22,23,24,25].

Recently, a more ‘systemic approach’ based on genomic tools has garnered significant attention in epilepsy management, aiming to tailor disease treatment. However, it is still debated whether the ketogenic diet (KD) can be considered a personalized medicine tool in the overall management of epilepsy [26,27]. As there are currently no specific treatment recommendations for patients with *PURA* syndrome [2], a structural comparison of wild-type and mutant proteins may provide insights into atomic-level structural differences caused by the genetic variation. Indeed, in the absence of experimental data, in silico genetic and protein analysis may help to improve our understanding of the response to pharmacologic and dietetic measures in patients resistant to first-line treatment and possibly predict alternative therapeutic options [28].

Our clinical data suggest that the positive response to the KD may depend on factors other than the mutation itself, possibly related to the ability of the *PURA* gene to elude the NMD process. The altered Purα protein undoubtedly plays a compromised transcriptional and translational role, although the predicted protein structure does not explain the improvement in the ketogenic diet approach. This aspect requires further investigation to determine whether there are non-canonical mechanisms within the cell that tolerate *PURA* transcripts with premature termination codons (PTCs), or if there are regulatory features upstream of the PTC that partially accomplish certain gene functions. Such mechanisms could potentially mitigate the clinical phenotype and lead to better treatment responses [19].

## 5. Conclusions

In our study, we demonstrated the usefulness of KD in the management of symptoms due to *PURA* syndrome, particularly in the control of sialorrhea and seizures in a young Italian patient with developmental disorder and epilepsy, who carries a novel frameshift pathogenic variant. We also examined the impact of the genetic variant on the protein structure, but a plausible relationship has not yet emerged due to the lack of information in the existing literature on patient outcomes of KD treatment.

Integrative analyses are recommended to collect clinical and molecular data to improve our understanding of the underlying mechanisms of disease variability and to evaluate more customized treatment strategies.

## Figures and Tables

**Table 1:** Ketogenic diet (KD) in patients with *PURA* syndrome: clinical and genetic characteristics.

Study	Gender	Genetics	Clinical Features	SZ Onset	SZ Types	SZ Outcome/ AED Response	KD Therapy	Adverse Effects of KD	FD
[1]	F	c.127-130delAGTG p.(Ser43Alafs*34) de novo	ID, H, nystagmus, AP, ST, CVI autism, mild pulmonic stenosis, CO, small hands and feet	3 y	FO, C	Unknown: LEV, OXC, LB	Unknown	-	Sialorrhea
	M	c.299T>G p.(Leu100Arg) de novo	ID, H, slow movements, central sleep AP, hypermetropia, small VSD, CO, retinoblastoma, small puffy hands and feet	3 y	MY	Unknown: CLB, LEV, RUF, VNS	Unknown	-	Sialorrhea
	M	c.697_699delTTC p.(Phe233del) de novo	ID, H, S, aberrant left subclavian artery. VSD, low vitamin D levels, high cholesterol, FD	3 y	LGS	Unknown: PER, CLB, RUF	Unknown	-	dysphagia
[2]	NR	c.98del p.(Gly33Alafs*45) de novo	Myoclonia, pyramidal tetraparesis spasticity of upper limbs, horizontal nystagmus, axial H osteoporosis	Day 3	MY, GTC	Sz reduction: LEV, PB No effect: VPA, ETX, RUF, LAC	No effect	-	-
	NR	c.159del p.(Leu54Cysfs*24) de novo	H, SC, delayed growth	2 y	Ab, FO, FO with sec. gen., A, reflex	Sz reduction: VPA (atonic sz only) No effect: CLB, TPM, LTG	No effect	-	-
	NR	c.289A>T p.(Lys97*) de novo	NA hypopituitarism, growth hormone deficiency	4 y	Ab, S	Sz free: CLB (transitory) Sz reduction: LTG, LAC, RUF Worsening: TPM, VPA	No effect	-	-
	NR	c.441delC; p. (Leu148Trpfs*77)	Ataxia, stereotypic hand movements, pyramidal and extrapyramidal symptoms, H, AP, SC, CO	12 y	FO, MY, A, T, GTC, reflex	Sz reduction; VNS, LTG, LEV No effect: LAC, PHT, CLB, CBD Worsening: VPA	Positive effect: sz reduction	-	-
	NR	c.451G>T p.(Glu151*) de novo	H	13 m	(FS at 6 m) GTC, S, FO with sec. gen.	Sz reduction: KD+TPM+CLZ No effect: ACTH, PB	Positive effect: sz reduction	-	FD (requiring G-tube)
	F	c.812_814delTCT p.(Phe271del) de novo	H, HS, ST, overgrowth, small muscular VSD, CO	6 m	S, T, C, West syndrome	Sz reduction: LEV (transitory), TPM (transitory), CLB No effect: VGB, ACTH, steroids, VPA	No effect	-	FD (requiring G-tube), GERD
[11]	F	c.586delA p.(Ile196SerfsTer29) de novo	Profound H left ventricular hypertrophic myocardiopathy due to septum enlargement, ST	Day 8	S, reflex (M), MY	Sz free with the addition of VPA to VGB	No effect	-	-
Present study	M	c.399_400dup; p.(Gln134ProfsTer92) de novo	H, epileptic SZ SC, delayed growth ST, CO, HY	1 m	S in flexion (30 episodes/ day) of the upper limbs and H of the neck with loss of consciousness	PH VPA: stopped due to adverse effects. LEV: no effect	Positive effects for sialorrhea Little improvement on SZ control.	Marked weakness exhaustion	Sialorrhea

Abbreviations: A: atonic, AA: atypical absences, Ab: absence, ACTH: adrenocorticotropic hormone, AP: apnea, CBD: cannabidiol, CBZ: carbamazepine, CLB: clobazam, C: clonic, CO: constipation, CVI: central vision impairment, CZP: clonazepam, ETX: ethosuximide, F: female, FO: focal, FEL: felbamate, FS: febrile seizure, FD: feeding difficulty, GTC: generalized tonic–clonic, HC: hemiclonic, H: hypotonia, HG: hypoglycemia, HS: hypersomnolence, KD: ketogenic diet, LAC: lacosamide, LEV: levetiracetam, LTG: lamotrigine, m: months, M: male; MY: myoclonic, NR: not reported, OXC: oxcarbazepine, PB: phenobarbital, PER: perampanel, PH: phenobarbital, PHT: phenytoin, RD: respiratory difficulty, RUF: rufinamide, S: spasms, sz: seizure, SC: scoliosis, ST: strabismus, T: tonic, TPM: topiramate, VGB: vigabatrin, VNS: vagal nerve stimulation, VPA: valproate, y: years, ZNS: zonisamide, symbol ‘-’: absent.

## Data Availability

All data generated or analyzed during this study are included in the published article.

## Supplementary Materials

The following supporting information can be downloaded at https://www.mdpi.com/article/10.3390/genes15070848/s1, Table S1: Summary of successfully produced protein models of Purα simulated by MODELLER v.10.1 (https://salilab.org/modeller/; last accessed on 15 April 2024). Highlighted in yellow is model number 4, as the most accurate prediction based on DOPE and GA341 scores, which was employed for the model reconstruction (Figure S1). These parameters are used for the discrimination of the best models. The range of the GA341 scores is 0.0 (worst) to 1.0 (native-like), while the lower the DOPE score, the better the model. *GA341 (Genetic Algorithm 341); DOPE (Discrete Optimized Protein Energy).* Supplementary Figure S1. In silico prediction of the effect of the patient variant on the protein structure. On the left, the 3D model of the crystal structure of human Purα (fragment Glu57-Glu212, PUR repeats I and II) from RCSB PDB (www.rcsb.org; last accessed on 15 April 2024); on the right, the model with frameshift variant p.(Gln134ProfsTer92) (c.399_400dup) of the *PURA* gene, showing the impaired protein folding of N-terminal and C-terminal PUR domains. Highlighted in red is the region where the frameshift mutation starts, and in yellow is the changed amino acid sequence.
