# Supplementary material for: PURA-Related Neurodevelopmental Disorders with Epilepsy Treated with Ketogenic Diet: A Case-Based Review
Source: Genes (Basel). 2024 Jun 27;15(7):848. doi: 10.3390/genes15070848 (PMC11276249; doi:10.3390/genes15070848)

Supplementary Materials: The following supporting information can be downloaded at: https://www.mdpi.com/article/10.3390/genes15070848/s1, Table S1: Summary of successfully produced protein models of Purα simulated by MODELLER v.10.1 (https://salilab.org/modeller/; last accessed on 2024-04-15). Highlighted in yellow is model number 4, as the most accurate prediction based on DOPE and GA341 scores, which was employed for the model reconstruction (Figure S1). These parameters are used for the discrimination of the best models. The range of the GA341 scores is 0.0 (worst) to 1.0 (native-like), while the lower the DOPE score, the better the model. GA341 (Genetic Algorithm 341); DOPE (Discrete Optimized Protein Energy). Supplementary Figure S1. In silico prediction of the effect of the patient variant on the protein structure. On the left, the 3D model of the crystal structure of human Purα (fragment Glu57-Glu212, PUR repeats I and II) from RCSB PDB (www.rcsb.org; last accessed on 2024-04-15); on the right, the model with frameshift variant p.(Gln134ProfsTer92) (c.399_400dup) of the *PURA* gene, showing the impaired protein folding of N-terminal and C-terminal PUR domains. In red highlighted is the region where the frameshift mutation starts, and in yellow is the changed amino acid sequence.

Table S1: Summary of successfully produced protein models of Purα simulated by MODELLER v.10.1 (https://salilab.org/modeller/; last accessed on 2024-04-15). Highlighted in yellow is model number 4, as the most accurate prediction based on DOPE and GA341 scores, which was employed for the model reconstruction (Figure S1). These parameters are used for the discrimination of the best models. The range of the GA341 scores is 0.0 (worst) to 1.0 (native-like), while the lower the DOPE score, the better the model. GA341 (Genetic Algorithm 341); DOPE (Discrete Optimized Protein Energy).

no.	DOPE	GA341
1	-12720.08984	1.00000
2	-12776.86621	1.00000
3	-12821.04492	0.99998
4	-12926.12891	1.00000
5	-12738.86719	1.00000
6	-12511.13867	1.00000
7	-12829.46289	1.00000
8	-12413.50488	1.00000
9	-12568.68262	1.00000
10	-12690.77051	1.00000
11	-12653.20703	1.00000
12	-12753.21582	1.00000
13	-12669.00684	1.00000
14	-12703.05859	1.00000
15	-12641.20312	1.00000
16	-12612.81055	1.00000
17	-12824.65039	1.00000
18	-12653.29395	1.00000
19	-12851.55371	1.00000
20	-12828.23633	1.00000

Supplementary Figure S1. In silico prediction of the effect of the patient variant on the protein structure. On the left, the 3D model of the crystal structure of human Purα (fragment Glu57-Glu212, PUR repeats I and II) from RCSB PDB (www.rcsb.org; last accessed on 2024-04-15); on the right, the model with frameshift variant p.(Gln134ProfsTer92) (c.399_400dup) of the *PURA* gene, showing the impaired protein folding of N-terminal and C-terminal PUR domains. In red highlighted is the region where the frameshift mutation starts, and in yellow is the changed amino acid sequence.

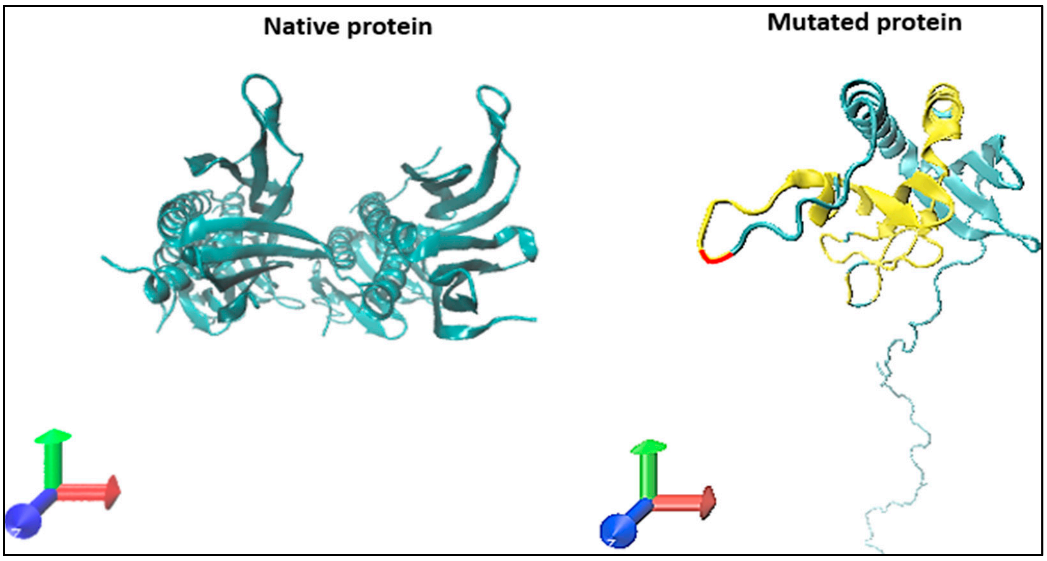

Supplement: Supplementary file 1 [file genes-15-00848-s001.zip › genes-3076132-supplementary.pdf]
